# Asymmetry of the Accessory Anterior Digastric Muscle Bellies: The Clinical Significance in Facial and Neck Surgery

**DOI:** 10.7759/cureus.7148

**Published:** 2020-02-29

**Authors:** Konstantinos Natsis, Maria Piagkou, Nikolaos Lazaridis, Nikolaos Anastasopoulos

**Affiliations:** 1 Anatomy and Surgical Anatomy, Aristotle University of Thessaloniki, Thessaloniki, GRC; 2 Anatomy and Surgical Anatomy, National and Kapodistrian University of Athens, Athens, GRC

**Keywords:** accessory muscle, anterior belly, digastric muscle, variation, hyoid bone, submental area, submandibular triangle

## Abstract

The anterior digastric muscle belly (ADMB) may present significant variations of substantial surgical importance. We present an unusual complex bilateral asymmetry of an accessory ADMB found when dissecting the submental area in a 72-year-old Greek male cadaver. A rare variant was recognized in the submental area constituted by a combination of bilateral asymmetry of the ADMB with unilateral absence of the intermediate tendon. The complex variant caused an obvious morphological asymmetry in the submental area. Such muscular variations may alter the surgical approach to the submental region. Clinicians involved in the treatment of this area should be aware of any possible variant, particularly when dealing with neck mass patients.

## Introduction

The digastric muscle (DM), located in the suprahyoid region, consists of an anterior and a posterior digastric belly (ADMB and PDMB), each of them arising from different embryological precursors and thus supplied by different nerves. The ADMB is innervated by the mylohyoid nerve (branch of the inferior alveolar nerve of the trigeminal nerve), while the PDMB is supplied by the facial nerve. Different innervation is explained by the separate derivation of the two bellies from the first and second brachial arch mesenchyme. The ADMB and PDMB are converged to the intermediate tendon (IMT) connecting with the hyoid bone (HB) through a fascial sling closely associated with the stylohyoid muscle (SHM) insertion. The ADMB originates from the digastric fossa. An aponeurotic layer gives off the tendon to the HB body and great horn. The DM may lack the IMT, thus be attached directly to the HB. The muscle is vascularized by the submental artery (branch of the facial artery) [1].

The ADMB presents a great variability in size, shape, and form. The ADMB variations may present double or extra slips spreading to the jaw or the mylohyoid muscle (MHM) or crossing over the midline to the opposite MHM or the opposite digastric fossa. These unilateral or bilateral muscle bundles or slips may occur as a result of a phylogenetic DM reduction or an unusual development of the ontogenetic material [2]. The ADMB is not identified in patients with hemifacial macrosomia and associated ipsilateral facial nerve palsy [3]. The accessory ADMBs may arise from the anterior belly itself, the IMT, the HB, the mandible or the digastric fossa and insert to the mylohyoid raphe, the HB, the mandible, the contralateral ADMB, or the MHM [4-7]. The variable DM formation may affect diagnostic and therapeutic procedures in head and neck surgery [2,8].

The current case report highlights the occurrence of a bilaterally asymmetrical accessory ADMBs and the unilateral absence of the IMT. The complex muscular variant caused an obvious asymmetry over the submental area. Knowledge of this kind of variability is of paramount importance in neck mass imaging interpretation and differential diagnosis. 

## Case presentation

**Figure 1:**
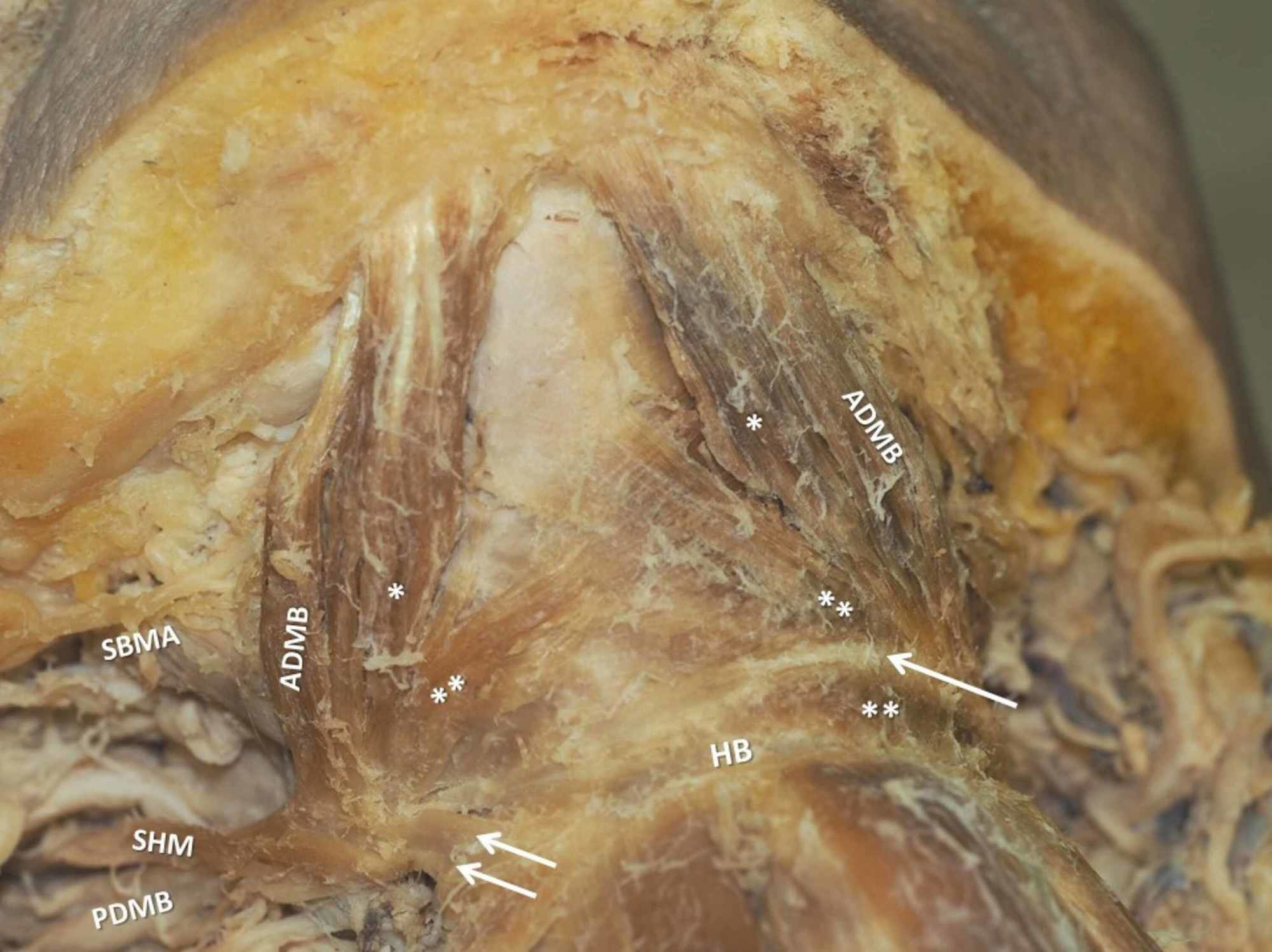
The asymmetrical anterior digastric muscle bellies (ADMB) and the accessory ones (*, **) Double white arrows indicate the right digastric muscle insertion. The single white arrow shows the fibrous band replacing the intermediate tendon. PDMB-posterior digastric muscle belly, SHM-stylohyoid muscle, SBMA-submental artery

**Figure 2:**
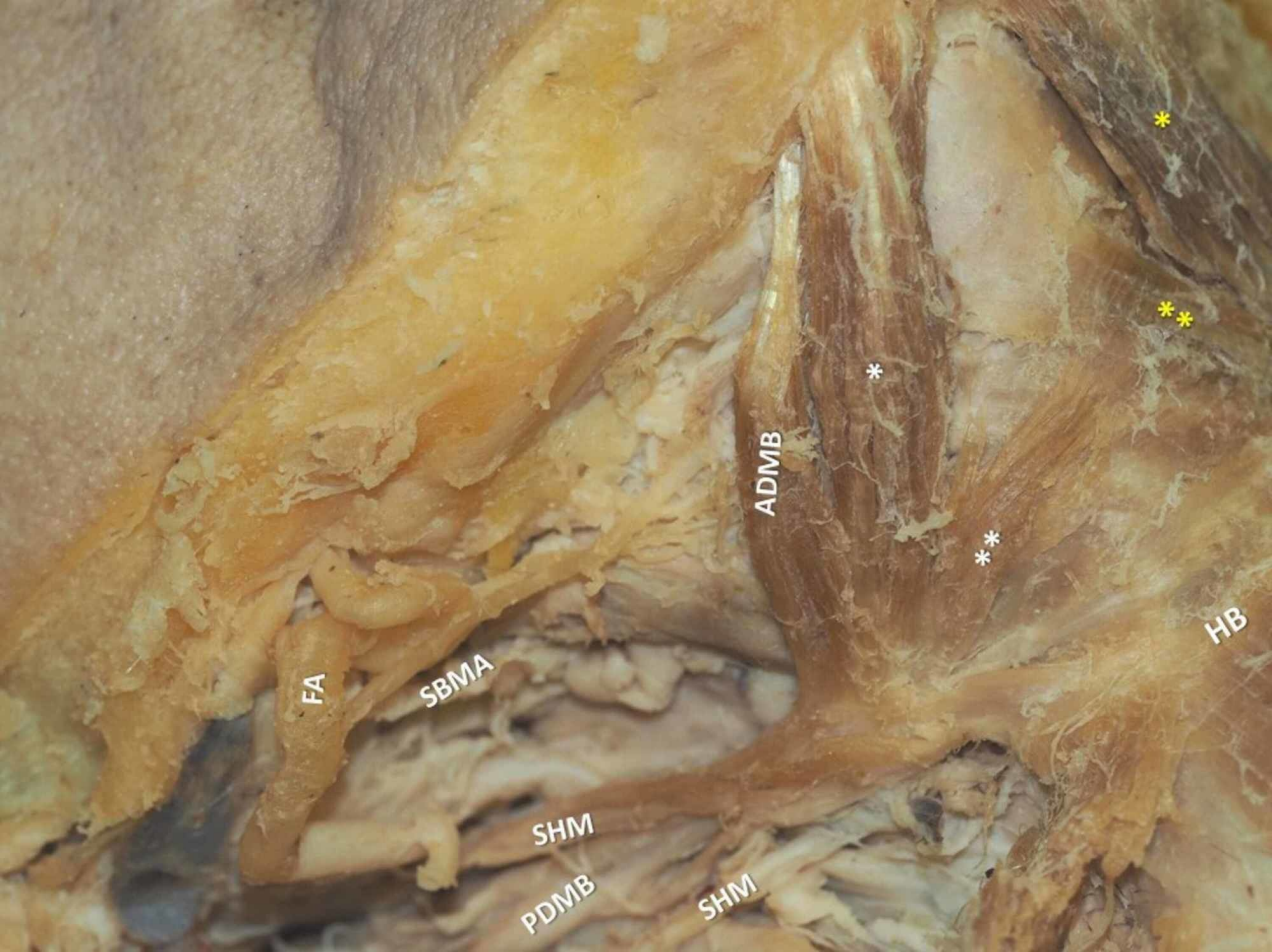
The right atypical anterior digastric muscle belly (ADMB, main belly) and accessory bellies (*, **) Yellow asterisks show the left ADMBs. FA-facial artery, SBMA- submental artery, SHM-stylohyoid muscle, PDMB-posterior digastric muscle belly, HB-hyoid bone

**Figure 3:**
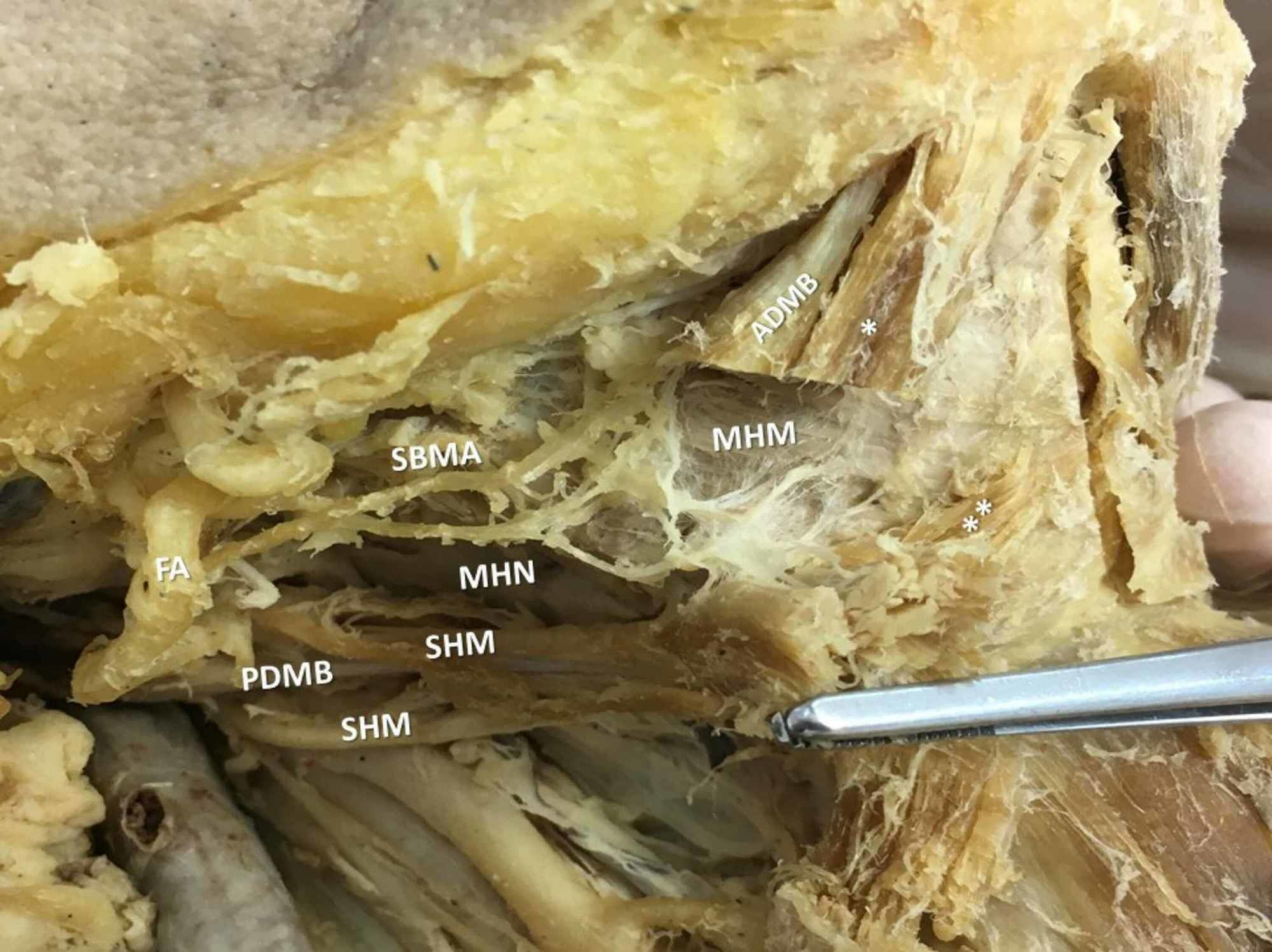
The dissected muscle MHN-mylohyoid nerve, FA-facial artery, ADMB-anterior digastric muscle belly, SHM-stylohyoid muscle, PDMB-posterior digastric muscle belly, SBMA- submandibular artery, * and ** - accessory bellies

During the submental region dissection in a 72-year-old Greek male cadaver, a bilateral accessory ADMB was observed (Figure 1). On the right side, the meticulous dissection revealed an SHM typically attached to the HB with an outer and an inner muscle bundles accompanying the IMT. The ADMB was constituted of three portions: a larger bulky ADMB atypically originated from the inferior mandibular rim, characterized as accessory belly; the main ADMB, smaller in size that typically originated from the digastric fossa; and a third thinner muscle bundle formed in the midline over the MHM raphe. All bundles ultimately merged at the IMT and attached to the HB through a strong fibrous band (Figures 2, 3). The submental artery and the mylohyoid nerve supplied the area.

**Figure 4:**
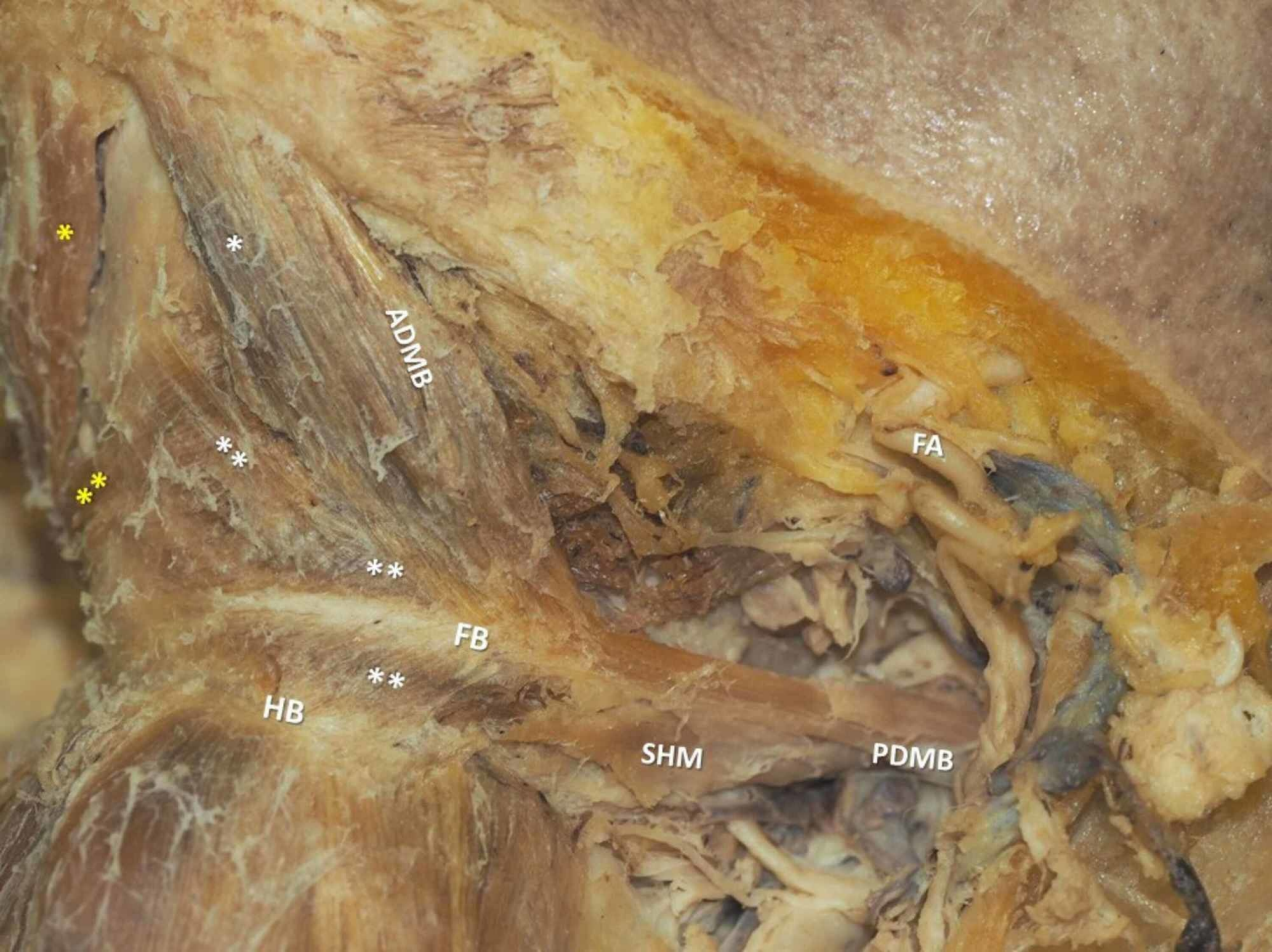
The left anterior digastric muscle belly (ADMB, main belly) and accessory bellies (*, **) The yellow asterisks show the right ADMBs. FA-facial artery, SHM-stylohyoid muscle, PDMB-posterior digastric muscle belly, HB-hyoid bone, FB-fibrous band, point of attachment of the pennated muscle fibers

Contralateral, the left SHM typically attached to the HB, via a single bundle, coursing along the inner aspect of the IMT and ending up into an extended strong fibrous band (representing an IMT extension in terms of function) without any attachment to the HB. Towards the superior and inferior aspect of the SHM, thin muscle bundles attached in a pennate fashion, the superior one ultimately crossed the midline over the MHM, while the inferior counterpart spread out freely over the HB. The main left-sided ADMB possessed two origins, one larger extended over the inferior mandibular rim, and a separate smaller one originating from the digastric fossa. Both parts merged further, forming a common belly and typically attached partially to the anterior end of the IMT and partially to the commencement of the fibrous band (Figure 4).

## Discussion

Embryological background of DM aberrations

Zlabek, studying the ADMB variants, found that the muscle expansion towards the midline was characterized by its rectangular oblongation [9]. In aberration cases, the variants were formed in conjunction with neurovascular development [9]. Holibkova and Machaiek reported the adaptive ADMB widening, laterally [8]. Symmetrical aberrations are extremely rare in human fetuses. Little is known about the mechanisms that guide the splitting of the muscle anlagen, although migration, fusion, displacement, and tissue interactions, as well as genetically programmed cell death, would be involved in definitive muscle formation. Accessory muscle formation may be attributed to an abnormal splitting of the muscle anlagen. The mylohyoid nerve supplies the ADMB and the MHM (derivates from the first pharyngeal arch), whereas the facial nerve supplies the PDMB and the SHM (derivates from the second pharyngeal arch). Abnormalities of the first and second pharyngeal arches during development may lead to multiple variations in the muscular complex ADMB-MHM and PDMB-SHM, respectively. This explains why these anomalies are coupled. Bilateral duplication of the ADMB may be due to a deficiency in the differentiation of the mesoderm of the first pharyngeal arch on both sides or to the abnormal migration of neural crest cells [2]. 

The ADMB variability among various studies

Several anatomical studies described the ADMB and DM fibrous sling variations [4,10]. The ADMB variants occur with an incidence ranging between 2.7% and 69.6% [3,5]. These aberrations are usually asymmetrical (20.7%) [6,11]. Mangalgiri and co-authors mentioned an equal incidence of unilateral and bilateral ADMBs variations in their sample [12]. Aktekin and co-authors described symmetrical variable ADMBs, in which the accessory bundles arranged in a cross, superficial to the MHM [13]. Celik and co-authors reported asymmetric accessory enlarged ADMBs extending from the HB to the chin [7]. In another study, Celik et al. referred that the muscle bands of a triple right-sided ADMB joined the IMT and expanded to the PDMB [14]. In another study, a unilateral quadrification of the ADMB was detected, while Sarikcioglu et al. reported an atypical DM with three accessory bellies and a fibrous band being inserted into the MHM raphe [10,15]. According to Sargon et al., the asymmetry in DM variants is more usual than symmetry [16]. Holibkova and Machaiek reported a combined asymmetrical variant with muscle bundles that unite the left digastric fossa with the IMT behind the contralateral ADMB [8]. There is usually some exchange of fibers sometimes amounting to complete fusion of the ADMB and the MHM [17]. An independent muscle, the mentohyoid (of Macalister) extending between the body of the HB and the mandibular symphysis, may be found along the ADMB medial border and could be considered as the third head of a trigastric muscle. Rarely, the ADMB may be absent or atrophied [3]. In some cases, no IMT exists and the PDMB may insert onto the styloid process, while the ADMB inserts onto the lateral side of the HB. The described case shows similarities with these findings. The ADMB may fuse with the MHM, at the MHM raphe. The fused part may form a separate accessory muscle connecting the DM with MHM [12,16].

Clinical significance

Asymmetrical or symmetrical ADMBs variations are of clinical importance since they indicate asymmetry cases over the anterior region of the neck or even in the movement of the floor of the mouth or the temporomandibular joint and perhaps imbalance in laryngeal movement [18]. The asymmetry may even be confounded in clinical and imaging examinations with submandibular or submental enlarged lymph nodes, benign cervical mass, or neoplasia [19].

The DM variability is of paramount importance during platysma myocutaneous flap mobilization in reconstructive procedures [19,20]. Thorough knowledge of the typical and variable ADMBs anatomy is of potential significance due to the muscle use in mouth reanimation after facial nerve palsy and submental artery flap procedures [20]. Also, the ADMB is used as a landmark during the lingual nerve or submandibular gland’s duct identification [13].

By using computed tomography (CT) and magnetic resonance imaging (MRI), typical and variable anatomic structures and pathologic entities of the floor of the mouth can be evaluated [5].

The described aberration represents a variant in the MHM-DM group in the floor of the mouth. This kind of ADMBs aberrations has to be taken into consideration when evaluating the floor of the mouth, as they can be confused with pathological conditions in imaging. Therefore, normal anatomical variants have to be considered on CT and MRI when an asymmetry over the floor of the mouth is encountered [11].

## Conclusions

Submental and submandibular areas are very well vascularized, and knowledge of their muscular abnormalities is essential when a myocutaneous flap is mobilized in reconstructive plastic surgery. Vascular variations may coexist, and in order to maintain and increase flap’s viability, vessels’ branching pattern must be well preserved, intraoperatively. CT and MRI are valuable imaging tools in the differential diagnosis of the muscular abnormalities from pathological lesions, as due to the muscular tissue density, accessory, and hypertrophied DM can be easily misinterpreted as a tumor or a lymph node. Cases of asymmetrical aberrancies in submental region should be very carefully identified in order to avoid misinterpretation.
